# Film Mulching with Straw Return and Controlled-Release and Conventional Urea Blend Alters Aggregate-Associated Nitrogen Distribution While Sustaining Grain Yield in Dryland Maize

**DOI:** 10.3390/plants15193044

**Published:** 2026-10-05

**Authors:** Fangfang Zhang, Yixuan Lv, Yanting Lu, Ziyi Song, Xuedong Chen, Kongyang Wu, Qilong Song, Renshan Li

**Affiliations:** 1 College of Life Science, Luoyang Normal University, Luoyang 471934, China; setzhang@126.com (F.Z.); 19930766043@163.com (Y.L.); 18638360792@163.com (Y.L.); 13782479599@163.com (Z.S.); chenxuedong1224@lynu.edu.cn (X.C.); kywu2007@lynu.edu.cn (K.W.); 2 State Key Laboratory of Soil and Water Conservation and Desertification Control, Northwest A&F University, Xianyang 712100, China; 3 College of Resources and Environmental Sciences, Gansu Agricultural University, Lanzhou 730070, China

**Keywords:** plastic film mulching, straw return, controlled-release and conventional urea blend, soil aggregates, aggregate-associated nitrogen, nitrogen uptake and translocation

## Abstract

Plastic film mulching (FM) improves crop productivity in dryland agriculture but risks soil quality degradation under continuous chemical fertilization. Under FM with optimized nitrogen (N) input from a controlled-release and conventional urea blend (CCU), straw return (SR) management may either contribute to grain yield or alter aggregate-associated N pools. A two-year film-mulched experiment was conducted with three treatments: no fertilization (CK), CCU alone (CU), and CCU plus SR (CS). Compared with CU, CS did not further increase grain yield despite greater total N uptake during critical growth stages. However, CS increased the proportion of large macroaggregate (LM) by an average of 10.0 percentage points and the mean weight diameter by 35.7%, and also increased N concentration in most aggregate fractions. A significant increase in N stock was detected in the LM fraction in both years. In contrast, N stock in the microaggregate (MI) was significantly lower under CS in 2020, whereas N stocks in small macroaggregate (SM), silt and clay (SC), and bulk soil were not significantly affected in either year. SM N stock was positively correlated with grain yield in the pooled dataset, but this association was attenuated after controlling for year and should therefore be considered exploratory. Overall, CS maintained grain yield comparable to CU while altering aggregate-associated N distribution, suggesting a potential decoupling between short-term yield and aggregate-associated N pools; however, the origin of the additional aggregate-associated N could not be resolved without isotopic tracing.

## 1. Introduction

Dryland agriculture supports global food production, yet crop productivity is constrained by water scarcity and ongoing soil degradation [1]. Sustaining dryland farming requires balancing grain yield against long-term soil fertility, which is commonly represented by soil organic carbon (SOC) and total nitrogen (TN) pools [2,3]. Historically, agronomic assessments often treat higher grain yield as the primary target, while soil health benefits may be overlooked [3]. This creates a potential trade-off or decoupling between immediate grain productivity and long-term soil nutrient sequestration [4]. Plastic film mulching (FM) combined with a single chemical fertilizer has been widely adopted in dryland agriculture to enhance crop yields by improving soil hydrothermal conditions [5]. However, long-term FM combined with sole chemical fertilization tends to deplete SOC and degrade soil structure [6,7]. Therefore, complementary management practices that rebuild soil organic matter (SOM) inputs are urgently needed for film-mulched dryland systems.

Straw contains abundant nutrients and contributes directly to soil carbon (C) and nitrogen (N) stocks when returned to the field [8]. China produces over 6.10 × 10^4^ Tg of crop straw annually [9]. Therefore, straw return (SR) is considered a promising practice for replenishing SOM and improving soil structure [4,10]. Previous studies have shown that SR can improve soil quality and crop yields by enhancing soil structure and nutrient cycling [4,10,11]. However, straw residues with high C/N ratios often induce microbial immobilization of mineral N and deplete soil moisture during the early decomposition phase, leading to transient N deficiency and suppressed crop growth [12,13]. To counteract this N immobilization, additional N fertilization is often required to synchronize N supply with crop demand. However, yield responses to SR are often absent when N fertilizer input is already optimized.

Solid granular urea (SGU) is the most widely used N fertilizer in maize production in Northwest China [14]. However, because SGU has a relatively short release period, it is generally recommended that split N applications be used to meet crop N demand throughout the growing cycle in global maize production [15,16,17]. In practice, implementing split N applications presents several challenges. These include higher labor and time costs for farmers, adverse weather conditions during critical growth stages, and insufficient machinery clearance to avoid damaging tall plants during the mid- to late operations [18]. Controlled release urea (CRU) can release N over an extended period, potentially addressing these challenges [14]. However, a one-time application of CRU is also subject to certain restrictions. It is relatively expensive, and its N release can be restricted by low temperatures or drought during early crop growth [18]. Therefore, one-time application of a mixture of SGU and CRU (hereafter CCU) can overcome the limitations of applying either fertilizer alone and better synchronize nutrient release with crop demand. In straw-returned systems, CCU may theoretically alleviate N immobilization while supplying crop N demand. However, previous studies have reported mixed yield responses to SR with N fertilizer, ranging from increases to no significant effects [19,20]. Most studies have focused on grain yield, with limited attention to physically protected N within soil aggregates.

Soil aggregation provides physical protection for soil organic matter and nutrients [21]. Macroaggregates (>0.25 mm) serve as key sinks for newly incorporated organic residues [22]. Although the individual effects of SR and N fertilization on aggregate formation are well documented, the quantitative relationships between crop N uptake, internal N partitioning, and N pools associated with aggregates remain poorly characterized under combined FM-CCU-SR management. Conceptually, it is unclear whether the additional N input from straw is preferentially allocated to grain or retained within vegetative organs and soil aggregates. However, addressing this issue directly would necessitate isotopic tracing (e.g., using ^15^N-labeled straw), a method that was not employed in the present study. Instead, our experiment quantifies differences in crop N uptake and N distribution associated with the treatments, providing evidence that is consistent with, but does not directly test, this hypothesis.

To address these knowledge gaps, a two-year field experiment was conducted in a film-mulched dryland spring maize system on the Loess Plateau. We hypothesized that, under FM with optimized N fertilization, the combination of SR and CCU would not further increase the grain yield compared to CCU alone. We further hypothesized that this treatment combination would alter N distribution among vegetative tissues and soil aggregates, consistent with a potential decoupling between short-term grain yield and aggregate-associated N distribution. Because N sources were not isotopically traced, we emphasize that these hypotheses concern treatment-associated N distribution patterns rather than the fate of straw-derived N per se. The specific objectives of the study were to (i) evaluate the effects of combined FM-CCU-SR management on crop N uptake, translocation and grain yield; (ii) quantify shifts in aggregate composition and aggregate-associated N stocks; and (iii) examine the associations between straw-N characteristics, crop N uptake and aggregate-associated N distribution.

## 2. Results

### 2.1. Soil Water Storage

**Figure 1:**
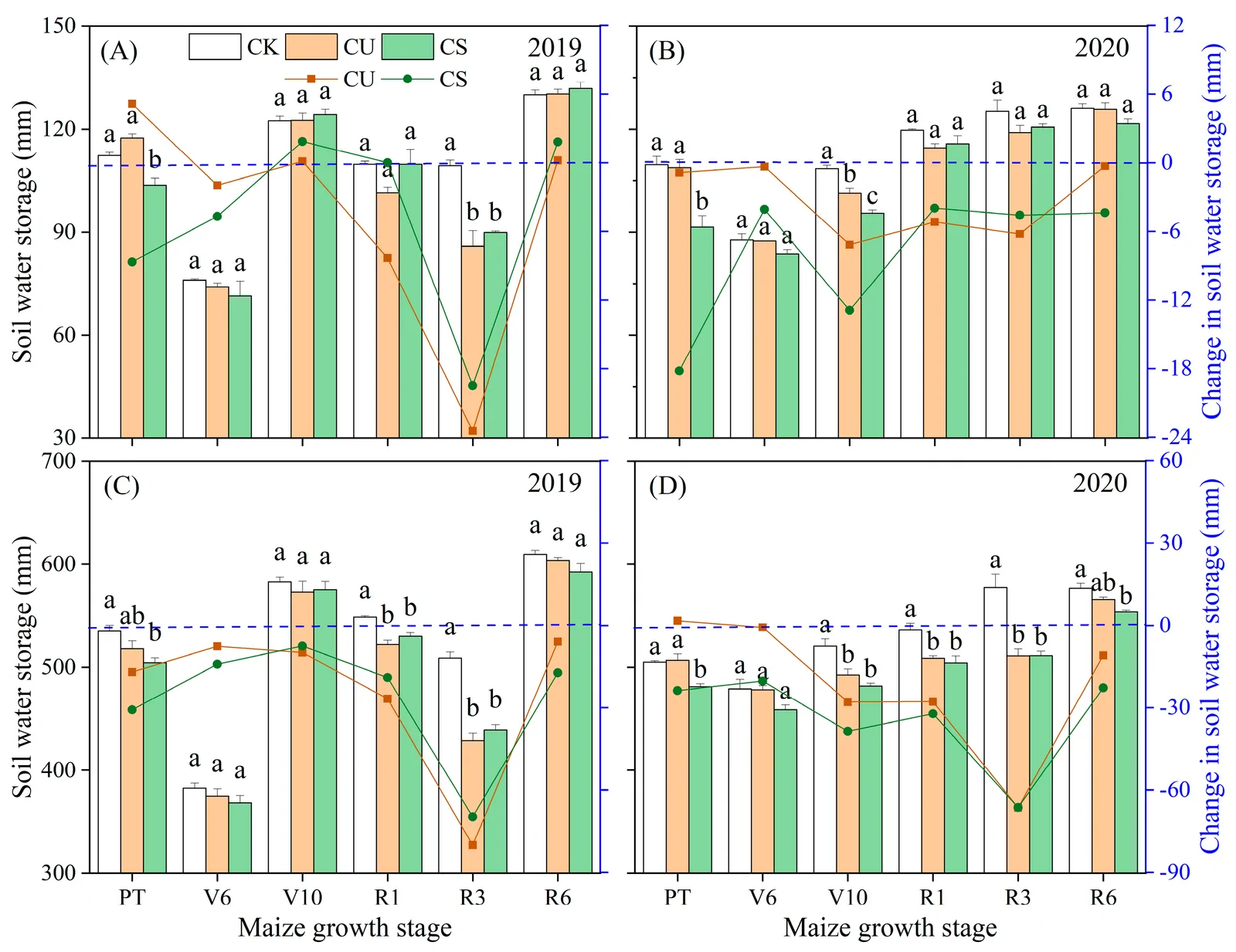
Dynamics of soil water storage and of the change in soil water storage in the 0–40 cm (**A**,**B**) and 0–200 cm (**C**,**D**) soil layers during the growing seasons in 2019 and 2020. Different lowercase letters indicate significant differences based on the LSD test (*p* < 0.05). The blue dashed line indicates that the change in soil water storage value relative to the control is zero. CK: FM + no fertilization; CU: FM + CCU; CS: FM + CCU + SR; PT: the pre-sowing stage; V6: the jointing stage; V10: the 10-leaf stage; R1: the silking stage; R3: the grain-filling stage; R6: the physiological maturity stage.

The SWS in the 0–200 cm soil layers over the two years was strongly influenced by precipitation, and that in the CU and CS treatments was generally lower than that in the CK at most sampling stages (Figure 1). The linear mixed-effects model revealed significant effects of treatment, year and growth stage on SWS in the 0–40 cm layer, with significant treatment × year, treatment × stage, and treatment × year × stage interactions (Table S3). For SWS in the 0–200 cm layer, treatment and growth stage effects were significant, while year and treatment × year effects were not significant; treatment × stage and treatment × year × stage interactions were, however, significant. These results indicate that the effects of SR on SWS varied with growth stage and, for the topsoil, with year. Compared with CK, CS significantly decreased SWS in the 0–40 cm soil layer at the PT and R3 stages in 2019 and at the PT and R1 stages in 2020 (Figure 1A,B). CU also significantly decreased SWS, but only at the R3 stage in 2019 and the R1 stage in 2020. In the 0–40 cm soil layer, the significant differences in SWS between CU and CS were observed only at the PT stage in 2019 and 2020. Similarly, compared with CK, CS significantly decreased SWS in the 0–200 cm soil layer at the PT, R1 and R3 stages in 2019 and at the PT, V10, R1, R3 and R6 stages in 2020 (Figure 1C,D). CU significantly decreased SWS at the R1 and R3 stages in 2019 and the V10, R1 and R3 stages in 2020.

Two consistent patterns emerged across both years. First, in terms of temporal dynamics, the difference in SWS between CU and CS was most pronounced during the early growth stages (PT and V6), but largely disappeared during the late growth stages (R3 and R6). The greatest difference in SWS between the two treatments occurred at the PT stage in both years, while the smallest differences were observed at the R3 stage in 2019 and the V10 stage in 2020. Second, in terms of vertical distribution, the effect of straw return on SWS was primarily confined to the top 0–40 cm soil layer. Weak and non-significant responses were detected in the deeper soil layers (40–200 cm) across all growth stages. No significant differences in SWS were observed between CU and CS in the 60–120 cm or 120–200 cm soil layers at any sampling stage in either year. In general, CS tended to increase water consumption in the early stages (PT and V6), but there was no significant difference in SWS between CU and CS in the later stages.

### 2.2. Grain Yield and Aboveground Dry Matter

#### 2.2.1. Grain Yield, Yield Components and Water Productivity

The results of grain yield, HI and WP for the CU and CS treatments are presented in Table 1. Compared with CU, there was no significant difference in grain yield under CS in 2019 and 2020. The number of cobs per hectare and kernels per ear followed similar trends over the two years. The 100-kernel weight under CS was slightly higher than that under CU in 2019 and 2020, but no significant difference was detected. HI is a commonly used indicator of assimilate partitioning to grain, but the difference in HI between the two treatments was not significant in 2019 and 2020. However, the WP under CS was slightly lower than that under CU in 2020, whereas it was marginally higher in 2019.

#### 2.2.2. Aboveground Dry Matter

**Figure 2:**
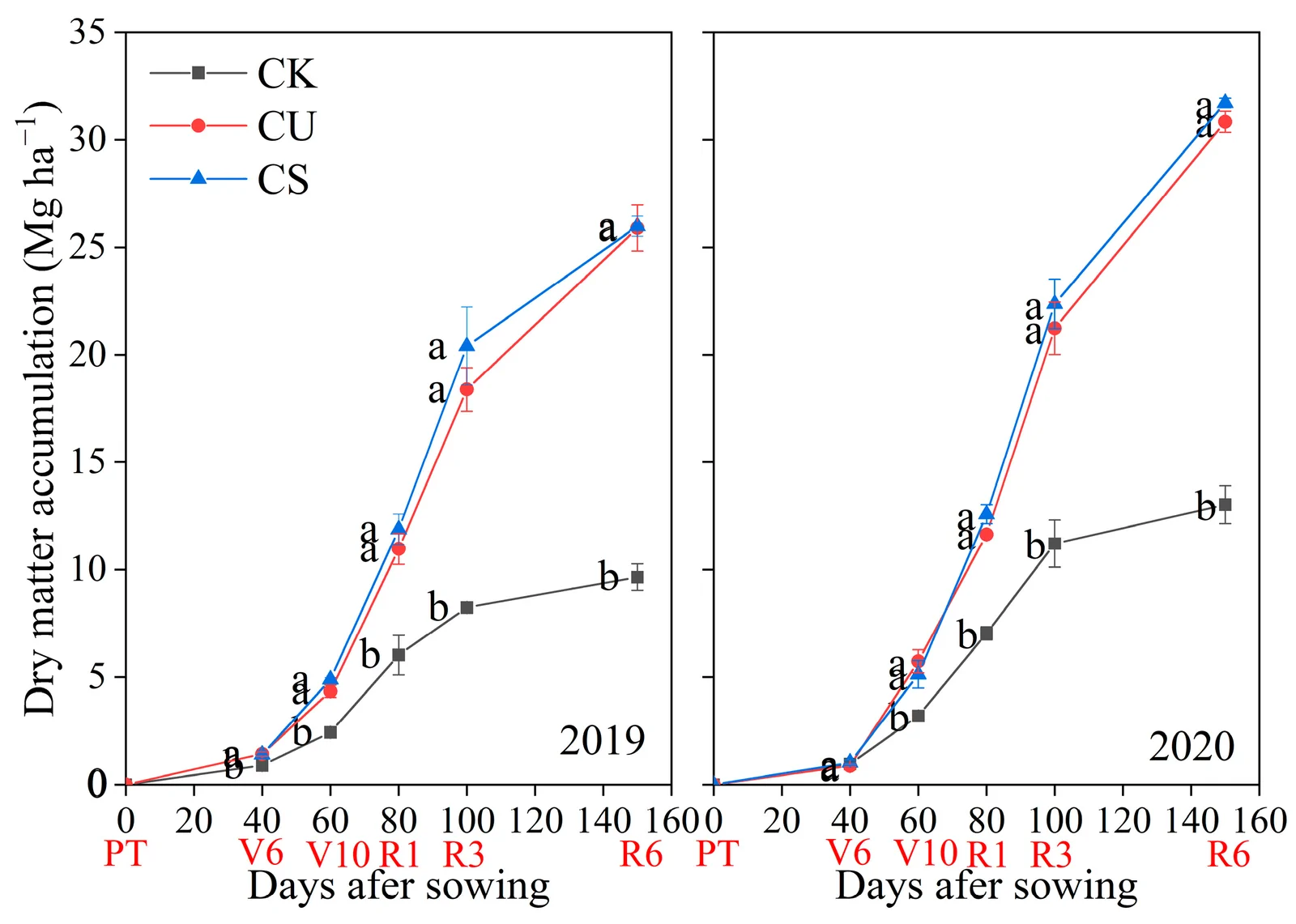
Dry matter accumulation curves of spring maize at different growth stages. Different lowercase letters denote significant differences among treatments in the same year at *p* < 0.05. CK: FM + no fertilization; CU: FM + CCU; CS: FM + CCU + SR; PT: the pre-sowing stage; V6: the jointing stage; V10: the 10-leaf stage; R1: the silking stage; R3: the grain-filling stage; R6: the physiological maturity stage.

The dynamics of aboveground dry matter accumulation followed a sigmoidal growth pattern for all treatments (Figure 2). The linear mixed-effects model showed that treatment, year, and growth stage significantly affected dry matter accumulation, with significant treatment × stage and treatment × year × stage interactions (Table S3). However, the treatment × year interaction was not significant, indicating that the treatment effect on dry matter accumulation was consistent across the two years. The highest dry matter accumulation rates were observed between V10 and R1, and between R1 and R3, in all treatments. In the control, it leveled off from R3 to R6, whereas CU and CS maintained relatively high rates during this period. All treatments reached their maximum dry matter content at R6. The dry matter accumulation under CU and CS was significantly higher than that of the control, with the exception of the V6 stage. The results indicate that CU and CS both significantly increased the accumulation of dry matter during all growth stages after the V6 stage of spring maize.

### 2.3. Nitrogen Uptake and Allocation in Spring Maize

#### 2.3.1. Aboveground Nitrogen Accumulation

**Figure 3:**
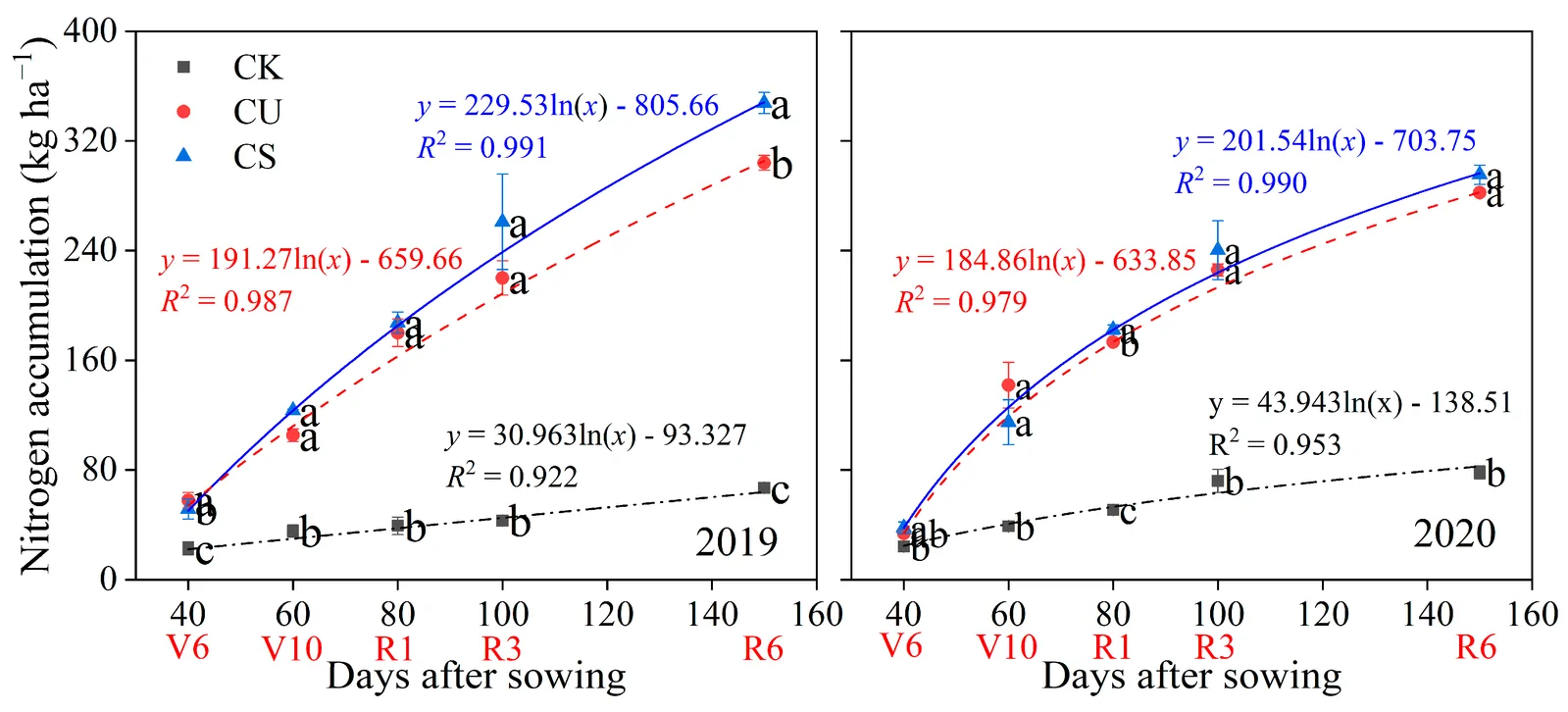
Aboveground N accumulation of spring maize at different growth stages and the fitting curve thereof. Different lowercase letters denote significant differences among treatments in the same year at *p* < 0.05. CK: FM + no fertilization; CU: FM + CCU; CS: FM + CCU + SR; PT: the pre-sowing stage; V6: the jointing stage; V10: the 10-leaf stage; R1: the silking stage; R3: the grain-filling stage; R6: the physiological maturity stage.

The temporal pattern of N accumulation paralleled that of dry matter accumulation. The N accumulation dynamics for spring maize in all treatments followed a logarithmic curve (Figure 3). For plant N accumulation, treatment and growth stage effects were highly significant, and the treatment × year interaction was also significant, while the year effect alone was not significant (Table S3). The three-way interaction was not significant. These results indicate that the treatment effect on plant N accumulation varied between years, but the overall pattern of N accumulation across growth stages was consistent. Compared with the control, CS significantly increased N accumulation, though the magnitude of the increase varied between years. In contrast, CU only significantly increased maize N accumulation after the V10 stage in both years. Notably, the differences widened significantly during the critical growth stage (R1 stage). CU and CS relative to CK showed 316% and 336% increases in N accumulation, respectively, at R1 in 2019 and 275% and 294%, respectively, at R1 in 2020. However, CS showed a significant difference in N accumulation compared with CU only at the R6 stage in 2020. These results suggest that both CU and CS significantly increased total N_up_ compared with CK during the growing season, but CS did not further increase total N_up_ beyond the levels achieved by the CU.

The N uptake by spring maize increased as the growth stages progressed under all three treatments (Figure 3). N uptake increased rapidly before the R1 stage, after which it slowed down. Growth trends for all treatments followed a logarithmic model. The logarithmic curve equations for the CU treatment in 2019 and 2020 were y = 143.81 ln(x) − 472.37 (R^2^ = 0.993) and y = 242.19 ln(x) − 860 (R^2^ = 0.997), respectively, and for the CS treatment were y = 160.32 ln(x) − 535.85 (R^2^ = 0.993) and y = 284.04 ln(x) − 1027.9 (R^2^ = 0.987) respectively.

#### 2.3.2. Nitrogen Translocation Proportion and Nitrogen Fertilizer Use Efficiency Indices

Both fertilized treatments significantly increased N translocation from vegetative organs to grains and improved N fertilizer use efficiency indices compared with CK (Table 2). However, no significant differences were detected between the CU and CS treatments. In both years, CU and CS exhibited significantly higher amounts of N translocation from the stem, leaf and bract compared with CK. Compared with CK, the stem N translocation under CU and CS was significantly increased by 24.5 and 30.9 kg ha^−1^ in 2019, respectively, and the corresponding values were 31.2 and 31.4 kg ha^−1^ for CU and CS in 2020, respectively. However, there was little difference between CU and CS across most organs and years. Compared with the control, CU and CS significantly increased the bract N translocation in both years, with no difference between them. The proportion of N remobilized ranged from 43.3% to 82.9% under CU and CS, with a higher remobilization efficiency observed in the stems. The proportion of grain N derived from vegetative organs ranged from 12.6% to 32.5% under CU and CS, with no consistent differences observed between the two. In terms of N utilization efficiency, CU and CS demonstrated similar performance across all indices. NAE ranged from 47.7 to 51.2 kg kg^−1^, NPE ranged from 67.0 to 70.4 kg kg^−1^, and NupE ranged from 0.98 to 1.63 kg kg^−1^. There were no significant differences between the CU and CS treatments in either year. Similarly, the NHI for CU and CS was significantly higher than for CK. NupE was substantially higher in 2020 than in 2019, whereas NAE, NPE and NHI were comparable to or slightly lower than in 2019.

The SPAD value increased significantly in CU and CS compared with CK during each growth stage in both years (Figure S2). The linear mixed-effects model, using the same specification as for SWS, dry matter, and plant N accumulation, revealed significant effects of treatment and stage on SPAD values, as well as significant treatment × year, treatment × stage, and treatment × year × stage interactions (Figure S2). The significant three-way interaction indicates that treatment effects on SPAD varied across both years and growth stages.

### 2.4. Soil Aggregate Distribution

#### 2.4.1. Water-Stable Aggregate Composition and Mean Weight Diameter

Both soil aggregate composition and MWD were significantly affected by SR over the two-year period (Table 3). In 2019, the proportion of the LM fraction was significantly higher, by 9.3 percentage points, under CS than under CU. There was no significant difference in the proportion of the SM fraction between the two treatments. Conversely, the proportion of the MI fraction under CS was significantly lower (13.5%) than that under CU (21.5%), while there was no significant difference in the SC fraction proportion between CU and CS. In 2020, the trends in aggregate composition were similar to those observed in 2019. The proportion of the LM fraction under CS was 10.7 percentage points higher than that under CU, while the proportions of the MI and SC fractions decreased by 12.7 and 5.1 percentage points, respectively. However, there was still no significant difference in the SM fraction proportion between the two treatments. Regarding the MWD, which reflects aggregate stability, CS exhibited substantial increases of 30.9% and 40.4% in 2019 and 2020, respectively. These results suggested that CS promoted the formation of large water-stable aggregates and significantly enhanced soil structural stability.

#### 2.4.2. Soil Aggregate-Associated Nitrogen

CS significantly affected the N concentrations in most aggregate fractions over the two-year period (Table 4). Compared with CU, CS significantly increased N concentrations in the SM and MI fractions by 0.07 and 0.07 g kg^−1^ in 2019, respectively. The CS treatment significantly increased N concentrations in the LM, MI and SC fractions by 0.15, 0.1 and 0.05 g kg^−1^ in 2020, respectively. However, there was no significant difference in N concentrations in bulk soil and SM fraction between the two treatments in 2020. Compared with CU, CS increased N concentrations in the bulk soil by 0.17 g kg^−1^ in 2019 and by 0.10 g kg^−1^ in 2020, although the latter difference was not significant. The results from both years showed that CS significantly increased N concentrations in most aggregate fractions, indicating that CS altered the distribution of N among aggregate size classes; an increase in N concentration alone, however, does not demonstrate an increase in total N retention, because the corresponding N stocks did not increase significantly for most fractions.

The ESM-corrected N stocks of each aggregate and bulk soil are presented in Table 4, and the uncorrected values are shown in Table S4. Over the two-year period, N stock in the LM fraction was significantly higher under CS than under CU in both years. Conversely, N stock in the MI fraction was significantly lower under CS than under CU in 2020. No significant differences in N stock were detected between CU and CS in the SM, SC fractions, or bulk soil in either year. Thus, while CS increased N concentrations in most aggregate fractions, significant increases in N stock were only detected in the LM fraction. The N stock in the bulk soil under CS was slightly higher than that under CU with no significant difference. In both treatments, the SM fraction accounted for over 50% of the TN stock in bulk soil over two years, which is consistent with its dominant role in aggregate mass distribution. However, compared with CU, CS increased the relative contribution of the LM fraction to the TN stock in bulk soil, while reducing the relative contribution of the MI fraction simultaneously. These results indicated a redistribution of aggregate-associated N toward the LM fraction at the expense of the MI fraction, with the LM fraction showing the greatest relative increase.

### 2.5. Principal Component Analysis

**Figure 4:**
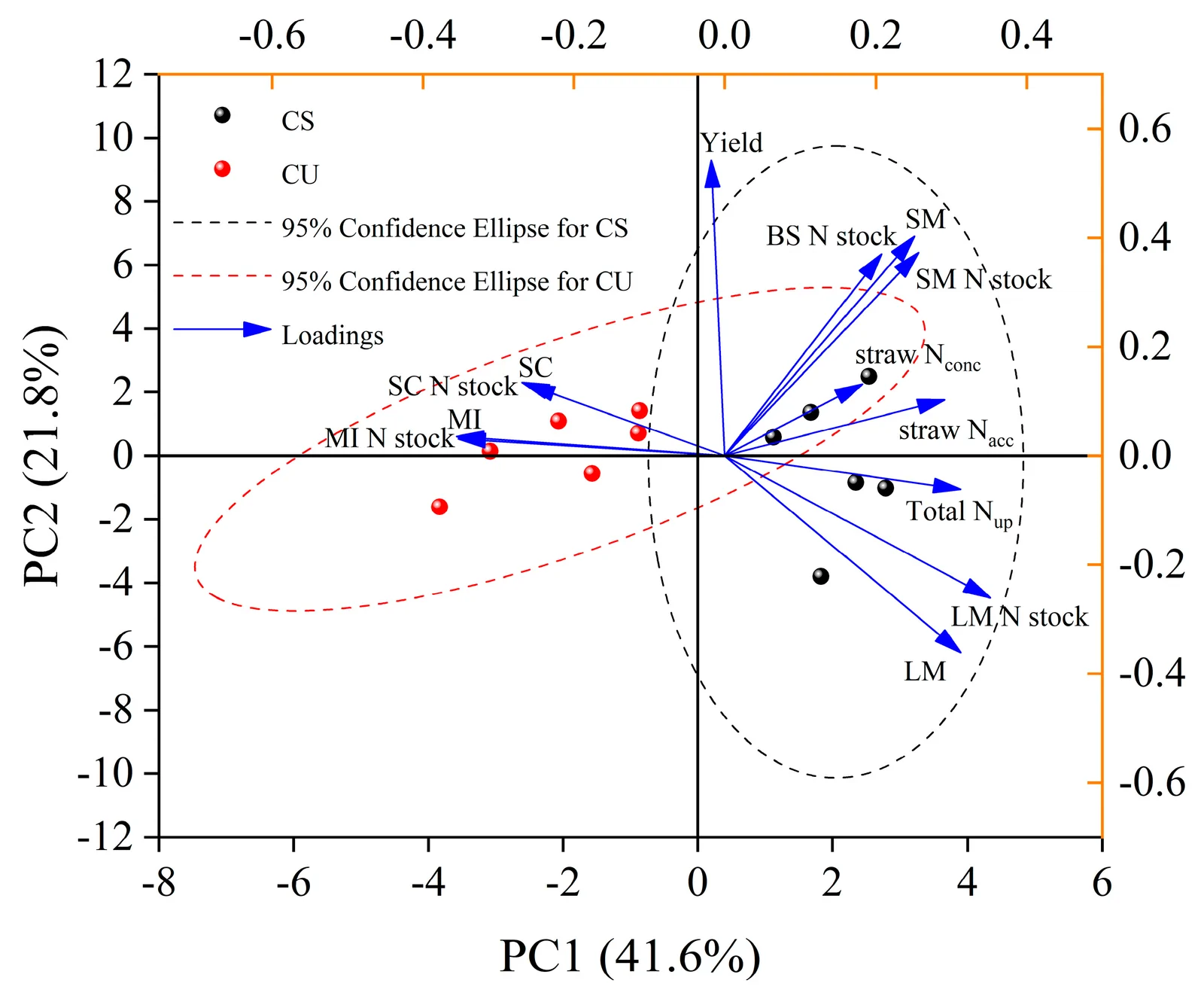
PCA of crop N parameters and soil aggregate N parameters affected by CU and CS treatments. PC1, principal component 1; PC2, principal component 2; CU: FM + CCU; CS: FM + CCU + SR; LM: >2 mm; SM: 0.25–2 mm; MI: 0.053–0.25 mm; SC: <0.053 mm; LM N stock: the N stock in LM fraction; SM N stock: the N stock in SM fraction; MI N stock: the N stock in MI fraction; SC N stock: the N stock in SC fraction; BS N stock: the N stock in bulk soil; Total N_up_: the total N uptake; straw N_conc_: the N concentration of the returned straw; straw N_acc_: the N accumulation of the returned straw.

PCA was performed to visualize the multivariate association patterns among crop N parameters and aggregate N parameters under CU and CS (Figure 4). The PCA revealed that the first and second principal components together explained 63.4% of the total variation. The PCA biplot showed a clear separation of CU and CS samples along PC1, with all CS replicates on the positive half-axis and all CU replicates on the negative half-axis, indicating distinct multivariate association patterns between the two treatments. Based on the loading vectors, SM, SM N stock, total N_up_, LM and LM N stock were closely associated with the positive side of PC1, which was characteristic of the CS treatment. Meanwhile, MI N stock, SC N stock, and MI and SC were associated with the negative side of PC1, which was characteristic of the CU treatment. The results indicated that the CS treatment was characterized by higher values of total N_up_ and macroaggregate-associated N parameters, whereas CU was characterized by higher values of finer aggregate fractions and their associated N parameters.

### 2.6. Relationship Between Aggregate Nitrogen Parameters and Crop Nitrogen Uptake as Well as Yield

**Figure 5:**
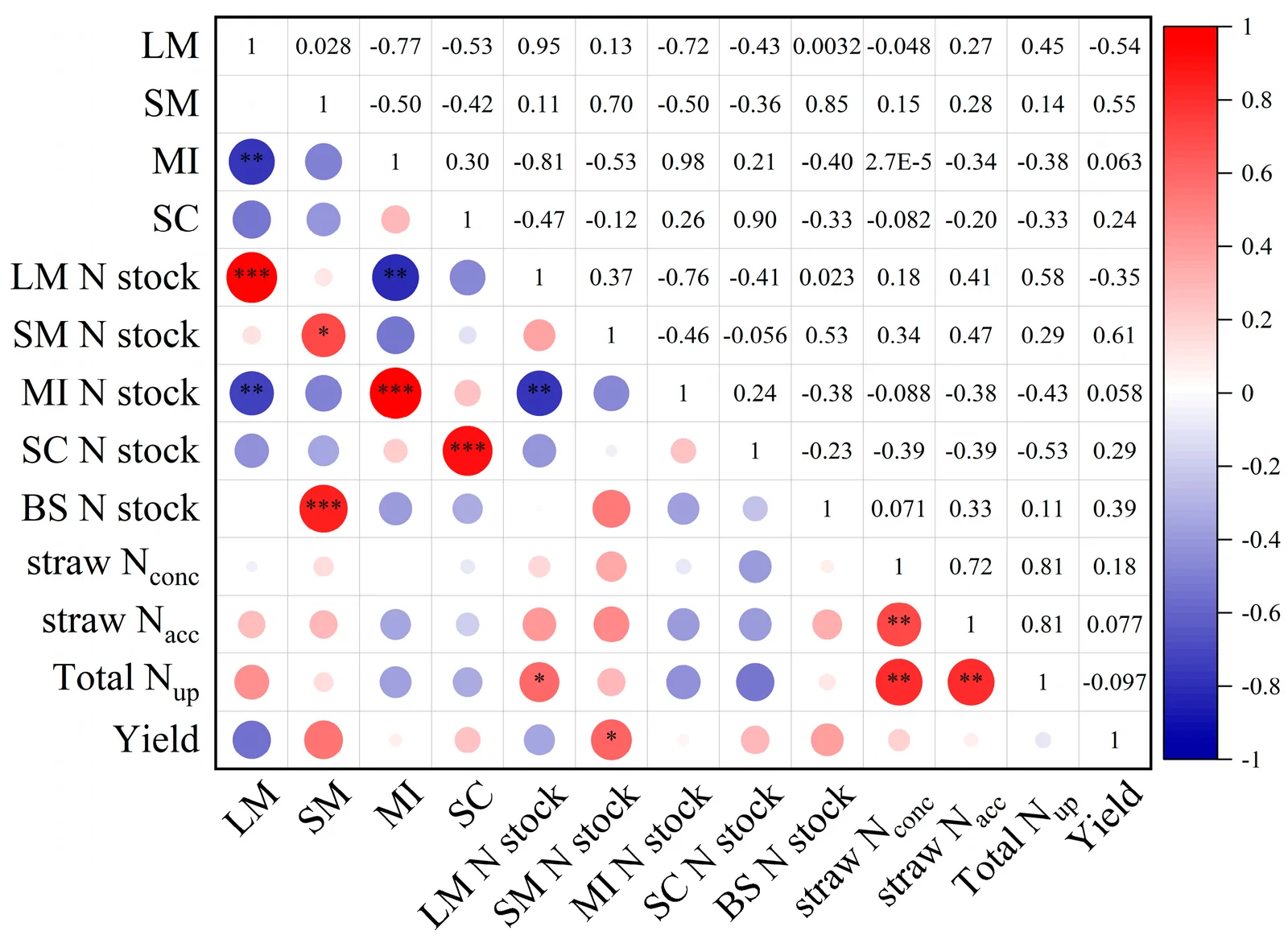
Pearson’s correlation coefficients between crop N parameters and soil aggregate N parameters under CU and CS treatments. CU: FM + CCU; CS: FM + CCU + SR; LM: >2 mm; SM: 0.25–2 mm; MI: 0.053–0.25 mm; SC: <0.053 mm; LM N stock: the N stock in LM fraction; SM N stock: the N stock in SM fraction; MI N stock: the N stock in MI fraction; SC N stock: the N stock in SC fraction; BS N stock: the N stock in bulk soil; total N_up_: the total N uptake; straw N_conc_: the N concentration of the returned straw; straw N_acc_: the N accumulation of the returned straw. The size of the circle indicates the strength of the correlation. *, **, *** indicate significant differences at *p* < 0.05, *p* < 0.01 and *p* < 0.001, respectively.

Pearson correlation analysis was performed to examine relationships among aggregate N parameters, straw N characteristics, total N_up_, and grain yield (Figure 5). It is important to distinguish between correlations that are mathematically constrained and those that reflect potentially independent biological relationships. The strong correlations between aggregate proportions and their corresponding N stocks are partly attributable to their shared calculation components and therefore serve primarily as internal consistency checks rather than independent evidence. Straw N_conc_ exhibited significant positive correlations with both total N_up_ (r = 0.811, *p* < 0.01) and straw N_acc_ (r = 0.719, *p* < 0.01). Additionally, total N_up_ demonstrated a significant positive correlation with straw N_acc_ (r = 0.805, *p* < 0.01). These three significant associations link straw N_conc_, total N_up_, and straw N_acc_. However, neither straw N_conc_ nor total N_up_ was significantly correlated with SM N stock (*p* > 0.05). In the pooled dataset, SM N stock was positively associated with grain yield (r = 0.610, *p* < 0.05). However, this relationship should be considered exploratory given the small and structured dataset, and causality cannot be inferred. LM N stock was also positively correlated with total N_up_ (r = 0.581, *p* < 0.05). However, there was no significant correlation between crop yield and straw N characteristics.

The results of the partial correlation analysis are presented in Table S5. Given that these observations arise from two treatments × two years × three blocks, we further performed partial correlation analyses to account for the structured nature of the data. After controlling for treatment, the association between SM N stock and grain yield remained significant (r = 0.801, *p* = 0.003). After controlling for year, the association was attenuated and became marginally non-significant (r = 0.596, *p* = 0.053). These results suggest that the SM N stock–yield association is not solely driven by treatment differences, but may be partly influenced by inter-annual variation. Therefore, this correlation should be considered exploratory, and its biological interpretation should be made with caution given the small and structured dataset. As shown in Table S5, the magnitude of this association varied considerably depending on whether year or treatment was controlled, further highlighting the statistical fragility of this relationship.

## 3. Discussion

### 3.1. Effects of Film Mulching with Straw Return and Controlled-Release and Conventional Urea Blend on Soil Water Dynamics

Numerous studies have demonstrated the effectiveness of FM in improving soil hydrothermal conditions and crop productivity [5,17]. CS tended to decrease topsoil SWC during early stages due to water consumption by decomposition, but increased it during post-silking stages, likely by reducing soil evaporative loss and improving infiltration of rainfall [23] (Figure S1). The effect of SR on SWS was most pronounced during the early growing stages and was largely confined to the topsoil layer, as also reported by Yang et al. [23]. The disappearance of treatment differences in later stages may be attributed to reduced soil BD and altered pore continuity in the topsoil under CS [23,24]. Although SWS under CS was numerically lower than under CU at some stages, the differences were not significant, indicating that straw return did not exacerbate soil water depletion relative to CU.

This study found that SWS was significantly lower under CU and CS than under CK at some stages (mainly R1 and R3), consistent with greater crop water use under N-fertilized treatments. However, SWS integrates multiple processes, and transpiration was not independently measured in this study. Therefore, the lower SWS under fertilized treatments cannot be attributed solely to transpiration. The difference between CU and CS in relative SWS change was largest at PT stage, which may be associated with water consumption during early straw decomposition, although this interpretation remains speculative because decomposition-related water use was not directly quantified [17]. In the second year, residual straw from the previous season may have decomposed more rapidly in early spring, temporarily competing with seedlings for water [17]. In later stages, the decomposing straw may have reduced bulk density and promoted root development and fungal networks, thereby enhancing aggregation and potentially improving water retention [25,26]. Overall, relative to CU, CS tended to increase topsoil SWC during post-silking stages, while SWS in the deeper profile remained comparable between the two treatments. Therefore, SR did not exacerbate soil water depletion, and its effects on soil water dynamics were largely confined to the topsoil layer during the early growing season.

### 3.2. Film Mulching with Straw Return and Controlled-Release and Conventional Urea Blend Maintained Yield but Enhanced Nitrogen Uptake Without Improving Nitrogen Fertilizer Use Efficiency

Grain yield is the primary criterion for evaluating agronomic practices in dryland farming [3]. Many studies have also shown that film mulching with SR can increase crop yield and soil water utilization [17,27,28]. In this study, both CU and CS significantly increased grain yield compared with CK in both years. The lack of yield improvement under CS compared with CU in our study is consistent with previous observations under optimized N rates [14,29]. However, positive yield responses have often been reported in N-deficient soils or long-term continuous SR experiments [4,30]. The crop response to nutrient supply under water-limited conditions is strongly dependent on water availability, highlighting the importance of considering water × nutrient interactions when interpreting maize yield responses in dryland environments [29]. There are two non-mutually exclusive explanations that may account for this absence of yield response. First, cold and dry winter conditions on the Loess Plateau may have delayed the straw decomposition, thereby limiting the release of nutrients within a single growing season [31]. Second, CCU may have supplied sufficient N to meet the crop’s demand throughout the season. Consequently, the relatively small amount of N mineralized from the straw may have been insufficient to produce an additional yield response [32]. Dry matter accumulation is closely linked to grain yield formation [33]. Previous studies have reported that SR can increase dry matter accumulation [17,34]. In line with these studies, CS showed slightly higher dry matter accumulation than CU, though this difference was not statistically significant. Overall, both CU and CS significantly increased crop yield and dry matter accumulation compared with CK, but CS did not differ from CU.

SR and CCU also affect N content in various plant organs and N uptake and translocation [30]. In this study, both CU and CS significantly increased stem and leaf N content at the silking stage compared with CK, with CS being numerically higher than CU. These findings are consistent with those of Guo et al. [35], who reported that a blend of controlled-release and conventional urea enhanced N translocation to grain. This resulted in increased N accumulation before silking and promoted N translocation from the vegetative organs to the grain in the fertilized treatments relative to CK. However, in the comparison between CS and CU, in line with the findings of Wang et al. [30], SR did not increase N translocation efficiency beyond that achieved with fertilizer alone. Grain N content at harvest was substantially higher under fertilized treatments than under CK (Table S2), consistent with the findings that fertilization increases grain N concentration [18,30]. SPAD values at R3 were comparable between CS and CU (Figure S2), indicating that leaf N status was not compromised under CS. Although this pattern is consistent with the interpretation that the additional N taken up under CS was not limiting for crop growth and was retained in non-grain tissues, our data do not demonstrate it. Because N sources were not traced isotopically and crop N demand was not assessed independently, this interpretation remains tentative. Studies have shown that N translocation to vegetative organs follows a quadratic response to increasing N application rates [30,36]. The N rate of 250 kg ha^−1^ used in this study was within the optimal range previously identified for this region [37,38,39]. Thus, at the optimal N rate, SR did not further increase N translocation to grain.

Consistent with the yield response, NAE, NPE, and NupE did not differ significantly between CU and CS (Table 2). The numerically lower NAE and NPE under CS may reflect dilution of fertilizer-derived N by straw-released N [40]. NHI was significantly higher under fertilized treatments than under CK, consistent with greater N remobilization efficiency at higher N supply levels [28]. Overall, CS maintained yield while enhancing N uptake comparable to CU, with the effect on N accumulation being more pronounced in 2020.

### 3.3. Film Mulching with Straw Return and Controlled-Release and Conventional Urea Blend Promotes Soil Aggregation and Alters Aggregate-Associated N Distribution

As the previous sections showed that CS increased N uptake but did not improve yield or N translocation efficiency, we examined whether the additional N input was associated with changes in soil aggregate N. Our study revealed that CS significantly increased the proportion of the LM fraction and decreased BD in the topsoil in both years (Table S1). However, the SM fraction showed no significant change (Table 3). This lack of change in the SM fraction is consistent with that of Wang et al. [4]. Similarly, CS significantly increased the MWD compared with CU in both years, indicating that it enhanced the soil aggregate stability of the topsoil. The increase in MWD may be associated with the binding effects of decomposing straw residues and changes in microbial activity, as reported in previous studies [11,26]. In addition, FM may contribute to aggregate stability by moderating soil moisture fluctuations [41]. In agreement with previous studies [4,42], the SM fraction accounted for the largest share of the bulk soil mass in both treatments, while the LM fraction showed the greatest relative increase under CS.

CS significantly decreased the N stock of the MI fraction (Table 4), even though MI N concentration was significantly higher under CS than under CU in both years. However, this reduction should not be interpreted as a loss of soil N, but rather as a redistribution of N among aggregate fractions driven by aggregate turnover. According to the hierarchical aggregate formation model [21,43], microaggregates bind together through organic binding agents, such as polysaccharides, fungal hyphae and root exudates derived from decomposing straw, to form larger macroaggregates. If this process occurred, the N associated with microaggregates would be incorporated into macroaggregates as they form. However, our study only measured aggregate distribution and N concentrations at harvest and did not track N movement among aggregate fractions. Therefore, while the observed pattern of a decrease in MI N stock accompanied by an increase in LM N stock (Table 4) and a significant negative correlation between MI proportion and LM proportion (r = −0.773, *p* < 0.01) is consistent with the hierarchical aggregate-formation hypothesis, it does not demonstrate the physical transfer of N from MI to LM. The reduction in MI N stock under CS may therefore reflect aggregate reorganization and changes in the distribution of N among aggregate fractions, rather than a net loss of soil TN. Isotope tracing or repeated sampling of aggregate fractions over time would be required to directly verify N movement among aggregate size classes.

The combined application of chemical fertilizer and SR has been reported to increase SOM and enhance nutrient turnover [4,40]. Long-term maize experiments likewise indicated that combining mineral fertilization with organic inputs can enhance crop nutrient acquisition while progressively improving soil fertility, emphasizing that agronomic responses should be evaluated together with changes in the soil nutrient pool [44]. Compared with CU, CS significantly increased N concentrations in most aggregate fractions and significantly increased N stock in the LM fraction in both years, while SM and bulk soil N stocks were not significantly affected (Table 4). This suggested that SR enhanced N concentration in aggregate fractions, which may contribute to long-term soil fertility [45]. These results suggested that SR may influence the redistribution of N among aggregate fractions, although the evidence for increased N stock was limited to the LM fraction in both years. Given the BD differences between treatments, these stock differences should be interpreted as indicative of N redistribution rather than as precise changes in soil TN storage. Nevertheless, the increased N concentration in aggregates under CS did not result in a higher yield, suggesting that the additional N associated with aggregates may not be immediately available for grain production. These findings indicated that CS was associated with changes in aggregate-associated N distribution, which may represent a more slowly cycling N pool compared with the bulk soil N.

### 3.4. Trade-Off Between Short-Term Grain Yield and Long-Term Soil Nitrogen Sustainability Under Film Mulching with Straw Return and Controlled-Release and Conventional Urea Blend

Maintaining crop productivity and preserving soil fertility are often viewed as competing goals in agricultural systems, driven by plant-microbial competition for available N [46,47]. Within our study system, CS produced grain yields comparable to CU without detectable yield penalties, implying no short-term trade-off between grain production and soil-aggregate responses under optimized N supply of CCU [48]. Compared with CU, CS significantly increased the proportion of LM in both years and LM N stock in both years, whereas SM N stock remained statistically unchanged (Table 3 and Table 4). The PCA results showed a separation of CU and CS samples along PC1, with SM N stock and LM N stock associated with the CS treatment and finer aggregate N parameters associated with the CU treatment. These multivariate patterns are consistent with the treatment-associated differences observed in the univariate analyses. However, although yield was not correlated with straw N characteristics or total N_up_, it showed a significant positive correlation with SM N stock (Figure S3). Partial correlation analysis further indicated that this association remained significant after controlling for treatment (r = 0.801, *p* = 0.003), but was attenuated after controlling for year (r = 0.596, *p* = 0.053), indicating that it was partly influenced by inter-annual variation. The significant variation in the correlation coefficient depending on whether year or treatment was controlled underscores the statistical fragility of this association (Table S5). Given the small sample size (n = 12) and the structured nature of the data, we refrain from drawing strong conclusions about the biological importance of this association. Although the exploratory SM N stock–yield association suggested a potential link between N associated with aggregates and yield, the lack of a yield response under CS, despite the fact that the SM N stock was numerically higher, suggested that the yield under CU had already reached a plateau.

Although the present measurements cannot resolve the underlying N fluxes, the correlations available here do not support a sequential transfer of straw N into aggregate N pools: straw N_conc_ was significantly correlated with total N_up_ (r = 0.811, *p* < 0.01), but neither straw N_conc_ nor total N_up_ was significantly correlated with SM N stock (*p* > 0.05). Because N sources were not traced isotopically, we cannot determine whether the additional N taken up under CS originated from straw, fertilizer, or pre-existing soil N, nor can we establish the direction of N transfer among pools. The absence of a significant increase in N translocation efficiency or NHI under CS relative to CU indicates that the additional N uptake was not accompanied by greater N remobilization to grain. The proposed sequence of straw N uptake, retention in vegetative tissues, and subsequent incorporation into aggregates should be regarded as a hypothesis to be tested with ^15^N tracing rather than a demonstrated pathway.

The observed benefits reported herein carry important implications for fertilization-strategy assessment in dryland farming systems [4]. In film-mulched agricultural systems focused primarily on grain-yield maximization, the soil-fertility benefits of straw return combined with CCU are easily overlooked if evaluations rely solely on grain yield. Our two-year observations suggested that this practice improves soil aggregation and alters aggregate-associated N distribution without yield penalties. The significant positive correlation between SM N stock and grain yield (r = 0.610, *p* < 0.05) indicates that aggregate-associated N and yield were positively associated in our dataset. However, this correlation does not establish that aggregate N is a causal determinant of yield, as both variables may respond to common factors such as treatment, year, or soil conditions. This highlights that soil health deserves consideration in agronomic management. Fertilization efficacy should therefore be evaluated using a multi-metric framework beyond grain yield, incorporating long-term soil-fertility indicators including aggregate stability, SOC pools and physically protected nutrient stocks [21,22,49]. This is particularly important in dryland regions, where soil degradation and fertility depletion remain ongoing threats to agricultural sustainability [1]. Overall, these two-year observations suggested that CS can modify the distribution of soil N among aggregate fractions without compromising short-term yield, a pattern that warrants further investigation through multi-year monitoring and ^15^N tracing.

Several limitations of this study should be acknowledged. First, the absence of ^15^N tracing prevents us from determining the origin of the additional aggregate-associated N, which could derive from straw, fertilizer, native SOM, or altered mineralization-immobilization processes. Second, the experimental design included only one N rate (250 kg ha^−1^), one SR rate (15 t ha^−1^) and one location. Because no SR without N fertilizer treatment or with alternative N rates treatments were included, the present design cannot separate the interaction between straw and CCU from the effect of SR under this particular N regime. Previous studies have shown that SR effects on soil aggregation and aggregate-associated N distribution can vary with N application rate [30,50], and that straw decomposition may be N-limited in the early phase [12]. Therefore, the extent to which our findings depend on the specific N regime (250 kg ha^−1^) remains an open question. Third, although treatments have been maintained since 2014, responses were measured only during 2019–2020. The observed differences likely reflect cumulative treatment effects, but their temporal dynamics remain unknown. Fourth, N-stock_FD_ calculations may be biased by treatment-induced differences in BD. Although ESM corrections were applied in this study, the resulting stock differences should not be interpreted as precise changes in soil TN storage, and repeated ESM-based assessments over time would be required to resolve temporal changes in soil N stocks. Fifth, several variables may be mathematically related or share common components, meaning that correlations among them may reflect shared calculation terms or common responses to treatment rather than independent biological processes. Additionally, the present study did not determine the microbial community, enzyme activities or risks associated with plastic-film residue, which limits our mechanistic interpretation. Nor were gaseous N losses (N_2_O, NH_3_) or long-term plastic residue accumulation quantified; the absence of a yield penalty should therefore not be read as a full “win-without-loss” assessment of the environmental balance.

## 4. Materials and Methods

### 4.1. Study Site Description

**Figure 6:**
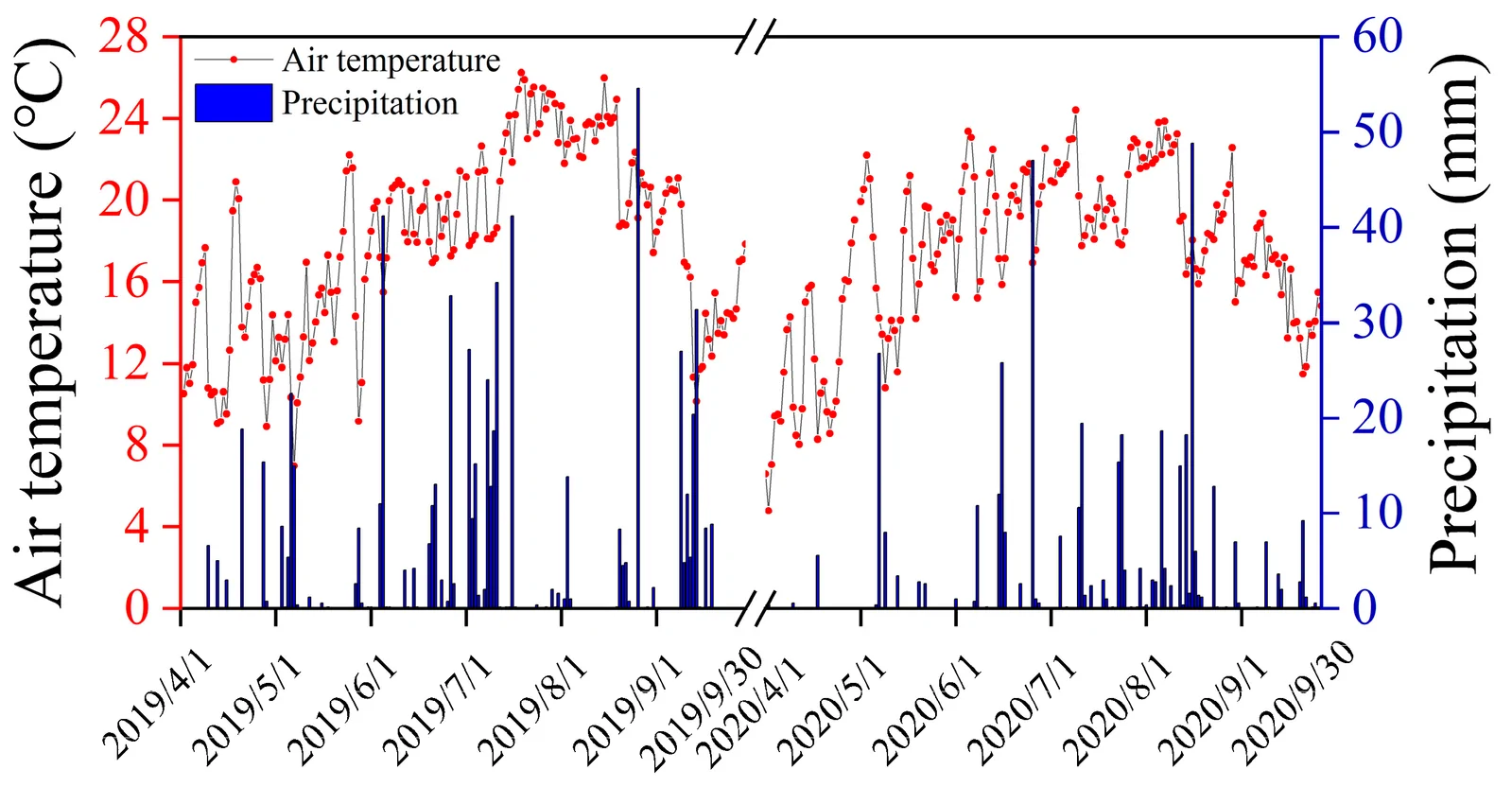
Monthly precipitation and mean air temperature at experimental site from April to September in 2019 and 2020.

The field experiment was established in 2014 at Changwu National Agricultural Ecological Experimental Station of Northwest A&F University (35°12′N, 107°40′E, 1200 m a.s.l.). It was located in the south-central part of the Loess Plateau with a warm temperate subhumid continental monsoon climate. The average annual temperature was 9.1 °C with a frost-free period of 171 days; the average annual rainfall was 584 mm, of which 73% fell from May to July each year. The annual evaporation was 1565 mm, and the groundwater table was more than 60 m deep. The soil type at the study site was a Cumuli-Ustic Isohumosol according to Chinese Soil Taxonomy, corresponding to a Calcic Kastanozem (WRB, 2022) and a Typic Haplustoll (USDA Soil Taxonomy). The daily precipitation and air temperature during the growing seasons in 2019 and 2020 are described in Figure 6. The basic chemical properties of the topsoil (0–20 cm) in April 2014 were: bulk density (BD, cutting-ring method) 1.33 g cm^−3^; particle-size distribution (laser diffraction) 37.9% sand, 40.7% silt and 21.4% clay (USDA textural class: loam); pH (1:2.5 soil/water suspension) 8.2; SOC (dichromate oxidation) 8.3 g kg^−1^; TN (Kjeldahl) 1.05 g kg^−1^; available phosphorus (Olsen) 21.5 mg kg^−1^; available potassium (NH_4_OAc extraction) 147.8 mg kg^−1^; and mineral N (KCl extraction) 21.6 mg kg^−1^ [51]. These values are thickness-weighted averages of the 0–10 and 10–20 cm layers, obtained from composite samples collected across the experimental field at establishment in April 2014.

### 4.2. Experimental Design

In this study, three treatments have been maintained in the same plots continuously since 2014, while the present study reports data collected during the 2019 and 2020 growing seasons. The three treatments were as follows: (1) FM + no fertilization (CK), (2) FM + CCU (CU), and (3) FM + CCU + SR (CS). Each treatment had three replicates and was arranged in a randomized complete block design. The plot area was 56 m^2^ (7 m × 8 m). The ridge-furrow system with full-film mulching involved alternating wide and narrow rows spaced 60 and 40 cm apart, which were covered with 0.008 mm thick non-biodegradable polyethylene film every year. The N application rates of the CU and CS treatments were each 250 kg ha^−1^, and SGU (N 46%) and CRU (N 44%, nutrient release period 70 days, Moith Inc.; Hefei, Anhui, China) were mixed in a 2:1 ratio (N from SGU: N from CRU) as the base fertilizer. All maize stalks (approximately 15 t ha^−1^) in the plots were chopped and incorporated into the topsoil after harvest. The rates of phosphate (P) and potash (K) fertilizer were the same for both treatments, 40 kg ha^−1^ of P in the form of calcium superphosphate (P_2_O_5_ 12%) and 80 kg ha^−1^ of K in the form of potassium sulfate (K_2_SO_4_ 51%). Both CU and CS treatments were fertilized only before sowing. The maize variety was Pioneer 335 with a sowing depth of 5 cm, and the density was 80,000 plants ha^−1^. During the experiment, the field moisture only came from precipitation.

### 4.3. Plant Sampling and Analysis

#### 4.3.1. Aboveground Biomass, Nitrogen Accumulation and SPAD

Maize plant development was identified using a standardized development stage system [52], and plant sampling was conducted using a standardized procedure to minimize subjective bias. At the jointing stage (V6), the 10-leaf stage (V10), the silking stage (R1), the grain-filling stage (R3) and the physiological maturity stage (R6), five consecutive plants were selected from the inner rows of each plot to avoid border rows and previously sampled areas; from these five plants, three representative plants were selected based on the following criteria: (i) plant height within ±10% of the plot mean, (ii) no visible symptoms of pest damage, disease or nutrient deficiency, and (iii) intermediate canopy vigor (neither the tallest nor the shortest, neither the most vigorous nor the weakest). The selected plants were then separated into organs (grain, stem, leaves, and others), oven dried at 105 °C for 30 min and subsequently at 75 °C until a constant weight was achieved, and weighed separately. Straw N concentration (straw N_conc_) was determined using the Kjeldahl method to calculate aboveground total N uptake (total N_up_).

For SPAD measurements, five adjacent plants were selected from each plot using the same selection criteria as described above. The SPAD value of the functional leaf (i.e., V6 and V10: the topmost fully expanded leaf; R1 and R3: the ear leaf) was measured using a portable chlorophyll meter (SPAD-502 Plus, Konica Minolta Inc.; Tokyo, Japan) [17]. Measurements were taken at the midpoint of each leaf blade, avoiding the midrib and leaf margins. Each leaf was measured three times and the average value recorded.

#### 4.3.2. Grain Yield and Water Productivity

At harvest in 2019 and 2020, the maize plants within 9 m^2^ (4.5 m × 2 m) were selected at the center of each plot to determine the grain yield, which was calculated with a standard moisture content of 15.5%. The number of kernels per ear was calculated by randomly selecting five maize cobs from each plot. A subsample of 1000 grains was air-dried to obtain the one thousand kernel weight. The harvest index (HI, %) was the ratio of the total kernel weight to the total aboveground biomass at R6.

The evapotranspiration (ET, mm) during the maize growing season for different treatments was calculated using a simplified water balance equation as follows [17].

(1) $$ET=P+({SWS}_{1}-{SWS}_{2})+C-R-D$$

where P (mm) is the precipitation during the maize growing season; SWS_1_ (mm) is the soil water storage at sowing; SWS_2_ (mm) is the soil water storage at harvest; C (mm) is the upward flow into the root zone; D (mm) is the downward drainage out of the root zone; and R (mm) is the surface runoff. The groundwater table at the experimental site was located approximately 60 m below the surface, so the upward flow into the roots was not considered in this area (C = 0). The experiment was conducted under rainfed conditions. No rainstorms occurred during the experimental period, and high ridges surrounded each plot to prevent runoff (R = 0). Local rainfall was generally low and did not cause underground drainage at depths exceeding 200 cm (D = 0).

WP (kg ha^−1^ mm^−1^) was calculated according to the following formula [53]:

(2) WP=Yield/ET

#### 4.3.3. Calculation of Nitrogen Translocation and Nitrogen Fertilizer Use Efficiency Indices

N translocation amount (NTA, kg ha^−1^), contribution of N translocation (CNT, %) and N translocation proportion (NTP, %) were calculated using the following equations [54].

(3) NTA = N accumulation in nutrient organs (at silking stage − at maturity stage)

(4) CNT = NTA/N accumulation in nutrient organs at silking stage × 100%

(5) NTP = NTA/N accumulation in grains at maturity × 100%

N fertilizer use efficiency indices included the N agronomic efficiency (NAE, kg kg^−1^), N partial factor productivity (NPE), N uptake efficiency (NupE) and N harvest index (NHI), which were calculated as follows [55]:

(6) NAE = (Yield of treatment − Yield of control)/N application rate

(7) NPE = Yield/N application rate

(8) NupE = Total N uptake/N application rate

(9) NHI = Grain N uptake/total N uptake

### 4.4. Soil Sampling and Analysis

#### 4.4.1. Soil Water Content and Soil Water Storage

Soil samples were collected at intervals of 20 cm from a 0 to 200 cm soil layer at the pre-sowing (PT), V6, V10, R1, R3 and R6 stages of spring maize. In each plot, three random soil core samples (each 4 cm diameter) were collected and mixed, then sealed in an aluminum case. The soil gravimetric water content (SWC, %) was measured by the oven drying method at 105 °C for 24 h (Figure S1). Soil BD was determined using the core method [56] (Table S1). The soil water storage (SWS, mm) was obtained by the conversion of SWC as follows [17]:

(10) SWS=SWC×BD×H×10

where SWC is the soil gravimetric water content at a specific soil layer (%), BD is the soil bulk density at a specific soil layer (g cm^−3^), and H is the soil depth increment (cm).

#### 4.4.2. Aggregate Fractionation and Determination of Nitrogen Concentration

Soil samples were collected from each plot using an auger at a depth of 0–20 cm at the harvesting stage each year. After air-drying, the collected soil samples were first sieved through an 8 mm sieve. For wet-sieving, air-dried soil samples were pre-wetted by capillary action to field capacity prior to analysis to avoid abrupt slaking. Water-stable aggregates were then determined using the wet sieving method [57,58]. The procedure separated four aggregate size classes: large macroaggregates (>2 mm; LM), small macroaggregates (0.25–2 mm; SM), microaggregates (0.053–0.25 mm; MI), and silt and clay (<0.053 mm; SC). After air-drying and grinding, the N concentration of aggregate fractions was determined using the Kjeldahl method [59]. The fixed-depth (FD) N stocks for a specific soil aggregate fraction were calculated as follows:

(11) $$i\;N\;{stock}_{FD}=P_{i}\times C_{i}\times BD\times 10$$

where i is a specific aggregate fraction, P_i_ is the proportion of fraction i (%), and C_i_ is the N concentration of the fraction i (g g^−1^).

As the BD differed between CU (1.40 g cm^−3^) and CS (1.32 g cm^−3^), the fixed-depth N stock estimates may be biased. To account for this, equivalent soil mass (ESM) corrections were applied using the approach of Ellert and Bettany (1995) [60]. Nevertheless, N stock calculations based on FD remain a limitation, and the resulting differences should not be interpreted as precise changes in soil TN storage.

(12) $$i\;N\;stock=(M_{ref}/M_{treatment})\times i\;N\;{stock}_{FD}$$

where N stock_ESM_ is the equivalent-soil-mass-corrected N stock of fraction i; N stock_FD_ is the fixed-depth N stock of fraction i; M_ref_ is the reference soil mass taken as the maximum soil mass among treatments (280 Mg ha^−1^ for CU, corresponding to a correction factor of 1.000); and M_treatment_ is the soil mass of the treatment being adjusted. The correction factor applied to CS was therefore 280/264 = 1.061; the same correction was applied to the bulk soil N stock.

The mean weight diameter (MWD) was calculated as follows [30]:

(13) $$MWD=\sum D_{i}\times W_{i}$$

where D_i_ is the mean diameter of fraction i (mm) and W_i_ is the proportion of fraction i (%).

### 4.5. Statistical Analyses

Data were processed using WPS Excel and analyzed with IBM SPSS Statistics 25.0 (SPSS Inc.; Chicago, IL, USA). Multiple comparisons were performed using the least significant difference (LSD) test at *p* < 0.05. Two types of statistical models were employed depending on the data structure: (1) For variables measured repeatedly across growth stages and years (SWS at 0–40 cm and 0–200 cm, aboveground dry matter accumulation, plant N accumulation and SPAD), linear mixed-effects models were fitted using the MIXED procedure, with treatment (T), year (Y), growth stage (S), and their interactions (T × Y, T × S, T × Y × S) as fixed factors, and block as a random factor to account for the randomized complete block design. The same model specification was applied to all four variables, including SPAD. Growth stage was treated as a repeated-measures factor within each plot. The covariance structure was selected based on the Akaike information criterion (AIC), and model convergence was verified for all final models. When the full random-effects structure (including random slopes) failed to converge, the random effects were simplified to a random intercept for plot (block was retained as a random blocking factor), which achieved convergence. Where treatment × stage or treatment × year × stage interactions were significant, treatment effects were compared within individual growth stages (and, where relevant, within individual years) using the LSD test; inference is therefore restricted to treatment comparisons within stages and years rather than to overall main-effect means. (2) For variables measured once per year (grain yield and yield components, ET, WP, N translocation parameters and N fertilizer use efficiency indices) and soil aggregate variables (aggregate size distribution, aggregate-associated N concentration and stock), two-way analysis of variance (ANOVA) was performed with treatment and year as fixed factors, and treatment × year as the interaction term. Block was retained as a random (blocking) factor in all models irrespective of its statistical significance, because the experiment followed a randomized complete block design. Pearson correlation analysis was performed on pooled data across treatments and years (n = 12). Because these observations arise from a structured design (2 treatments × 2 years × 3 blocks), partial correlation analyses were additionally conducted to control for the effects of treatment and year separately. All correlation analyses should be considered exploratory given the limited sample size. These coefficients are descriptive: they quantify pairwise association only and cannot establish causal relationships, mediation or the direction of N transfer among pools. Principal component analysis (PCA) was used as a descriptive ordination to visualize multivariate association patterns, rather than as a test of treatment effects. In all analyses, *p* < 0.05 was considered statistically significant. All graphs were drawn using OriginPro 2021 (OriginLab Inc.; Northampton, MA, USA).

## 5. Conclusions

Under the conditions of this two-year assessment within a long-term field experiment, our results showed that CS maintained maize grain yield comparable to CU while increasing macroaggregate formation, MWD, and N concentration in several aggregate fractions. A significant increase in N stock under CS relative to CU was detected in the LM fraction in both years, whereas N stocks in the SM, SC and bulk-soil fractions were not significantly affected. Given the BD differences between treatments, these stock differences should be interpreted as indicative of N redistribution rather than as precise changes in soil TN storage. Meanwhile, grain yield was not correlated with straw N characteristics or total N uptake, but showed a positive correlation with SM N stock in the pooled dataset. Partial correlation analysis indicated that this association remained significant after controlling for treatment, but was attenuated after controlling for year, suggesting that the relationship is exploratory and may be partly influenced by inter-annual variation. This association should therefore not be elevated to mechanistic evidence. These responses indicate that SR can alter the distribution of N among aggregate fractions without compromising short-term productivity. However, because N sources were not isotopically traced, the contribution of straw-derived N to plant uptake and aggregate-associated N pools cannot be quantified, and the proposed pathway of straw N transfer into aggregates remains hypothetical. Moreover, longer-term measurements are required to determine the persistence of these responses. Future research should employ multi-year monitoring and ^15^N tracing to quantify the contribution of straw-derived N to soil N pools under different N regimes.

## Figures and Tables

**Table 1:** Grain yield, total cobs per hectare, kernel number per ear, 100-kernel weight, HI, ET and WP of spring maize under different treatments at maturity stage in 2019 and 2020.

Year	Treatment	Grain Yield	Cobs	Kernels	100-Kernel Weight	HI	ET	WP
-	-	Mg ha^−1^	No. ha^−1^	No./ear	g	%	mm	kg ha^−1^ mm^−1^
2019	CK	4.81 ^b^	72,222 ^b^	351 ^b^	24.6 ^b^	30.2 ^b^	540 ^a^	8.9 ^b^
	CU	17.6 ^a^	84,444 ^a^	615 ^a^	33.2 ^a^	68.1 ^a^	531 ^a^	33.2 ^a^
	CS	17.5 ^a^	86,296 ^a^	634 ^a^	33.7 ^a^	67.5 ^ab^	525 ^a^	33.5 ^a^
2020	CK	4.85 ^b^	75,185 ^b^	259 ^b^	21.0 ^b^	42.8 ^a^	348 ^a^	13.8 ^b^
	CU	17.5 ^a^	84,444 ^a^	658 ^a^	28.1 ^a^	53.5 ^a^	347 ^a^	50.4 ^a^
	CS	16.8 ^a^	82,963 ^ab^	647 ^a^	28.4 ^a^	50.2 ^ab^	347 ^a^	48.3 ^a^
Source of variation								
Year (Y)		ns	ns	ns	**	**	***	***
Treatment (T)		**	ns	ns	ns	ns	ns	ns
Y × T		ns	ns	ns	ns	ns	ns	ns

Different lowercase letters denote significant differences among treatments in the same year at *p* < 0.05. **, ***, ns indicate significant differences at *p* < 0.01 and *p* < 0.001, and non-significance, respectively. CK: FM + no fertilization; CU: FM + CCU; CS: FM + CCU + SR.

**Table 2:** Effects of different treatments on nitrogen accumulation, partitioning and uptake in spring maize organs at physiological maturity in 2019 and 2020.

Year	Treatment	N Translocation Amount			N translocation Proportion			Contribution of N Translocation			NAE	NPE	NupE	NHI
-		kg ha^−1^			%			%			kg kg^−1^	kg kg^−1^	kg kg^−1^	%
-	-	Stem	Leaf	Bract	Stem	Leaf	Bract	Stem	Leaf	Bract	-	-	-	-
2019	CK	11.1 ^b^	9.6 ^b^	3.7 ^b^	67.6 ^a^	50.9 ^b^	69.9 ^a^	22.6 ^a^	19.7 ^b^	7.4 ^a^	-	-	-	71.7 ^b^
	CU	35.6 ^a^	60.0 ^a^	19.1 ^a^	67.6 ^a^	67.6 ^a^	82.9 ^a^	19.2 ^a^	32.5 ^a^	10.4 ^a^	51.2 ^a^	70.4 ^a^	0.98 ^a^	76.6 ^a^
	CS	42.0 ^a^	61.5 ^a^	17.1 ^a^	70.1 ^a^	66.6 ^a^	81.9 ^a^	22.2 ^a^	32.3 ^a^	9.0 ^a^	50.9 ^a^	70.2 ^a^	1.01 ^a^	76.7 ^a^
2020	CK	4.5 ^b^	7.2 ^b^	2.4 ^b^	25.1 ^b^	24.7 ^b^	42.4 ^b^	14.5 ^a^	22.6 ^a^	7.4 ^a^	-	-	-	40.9 ^b^
	CU	35.7 ^a^	53.5 ^a^	9.9 ^a^	53.6 ^a^	49.5 ^a^	48.9 ^a^	14.5 ^a^	21.8 ^a^	3.9 ^a^	50.7 ^a^	70.1 ^a^	1.55 ^a^	68.9 ^a^
	CS	35.9 ^a^	47.8 ^a^	15.1 ^a^	54.1 ^a^	43.3 ^a^	51.2 ^a^	12.6 ^a^	16.6 ^a^	5.2 ^a^	47.7 ^a^	67.0 ^a^	1.63 ^a^	68.0 ^a^
Source of variation														
Year (Y)		ns	ns	**	ns	ns	***	ns	ns	**	ns	ns	***	ns
Treatment (T)		ns	ns	ns	ns	ns	ns	ns	ns	ns	ns	ns	**	ns
Y × T		ns	ns	ns	ns	ns	ns	ns	ns	ns	ns	ns	*	ns

Different lowercase letters denote significant differences among treatments in the same year at *p* < 0.05. *, **, ***, ns indicate significant differences at *p* < 0.05, *p* < 0.01 and *p* < 0.001, and non-significance, respectively. CK: FM + no fertilization; CU: FM + CCU; CS: FM + CCU + SR; NAE: N agronomic efficiency; NPE: N partial factor productivity; NupE: N uptake efficiency; NHI: N harvest index.

**Table 3:** Mean distribution of soil aggregate fractions in the topsoil under CU and CS treatments.

Treatments	LM	SM	MI	SC	MWD
-	%	%	%	%	mm
2019					
CU	16.3 ^b^	50.8 ^a^	21.5 ^a^	11.3 ^a^	1.43 ^b^
CS	25.6 ^a^	52.4 ^a^	13.5 ^b^	8.8 ^a^	1.89 ^a^
2020					
CU	17.3 ^b^	47.7 ^a^	23.6 ^a^	12.1 ^a^	1.44 ^b^
CS	28.0 ^a^	53.8 ^a^	10.9 ^b^	7.0 ^b^	2.02 ^a^
Source of variation					
Year (Y)	ns	ns	ns	ns	ns
Treatment (T)	**	ns	***	ns	***
Y × T	ns	ns	ns	ns	ns

Data are presented as the means (n = 3). Different lowercase letters denote significant differences among treatments in the same year at *p* < 0.05. **, ***, ns indicate significant differences at *p* < 0.01 and *p* < 0.001, and non-significance, respectively. CU: FM + CCU; CS: FM + CCU + SR; LM: large macroaggregate; SM: small macroaggregate; MI: microaggregate; SC: silt and clay; MWD: mean weight diameter.

**Table 4:** N concentration and equivalent-soil-mass corrected N stock of soil aggregate fractions and bulk soil under CU and CS treatments.

Treatments	Aggregate N Concentration (g kg^−1^)					Aggregate N Stock (Mg ha^−1^)				
	LM	SM	MI	SC	BS	LM	SM	MI	SC	BS
2019										
CU	1.20 ^a^	1.05 ^b^	0.88 ^b^	0.74 ^b^	0.95 ^b^	0.55 ^b^	1.50 ^a^	0.53 ^a^	0.24 ^a^	2.67 ^a^
CS	1.31 ^a^	1.12 ^a^	0.95 ^a^	0.79 ^a^	1.12 ^a^	0.93 ^a^	1.64 ^a^	0.36 ^a^	0.20 ^a^	3.14 ^a^
2020										
CU	1.01 ^b^	1.02 ^a^	0.95 ^b^	0.97 ^a^	0.95 ^a^	0.49 ^b^	1.37 ^a^	0.63 ^a^	0.33 ^a^	2.65 ^a^
CS	1.16 ^a^	1.05 ^a^	1.05 ^a^	1.02 ^a^	1.05 ^a^	0.91 ^a^	1.57 ^a^	0.32 ^b^	0.20 ^a^	2.92 ^a^
Source of variation										
Year (Y)	**	**	***	***	ns	ns	ns	ns	ns	ns
Treatment (T)	**	**	***	*	*	**	ns	**	ns	ns
Y × T	ns	ns	ns	ns	ns	ns	ns	ns	ns	ns

Different lowercase letters denote significant differences among treatments in the same year at *p* < 0.05. *, **, ***, ns indicate significant differences at *p* < 0.05, *p* < 0.01 and *p* < 0.001, and non-significance, respectively. CU: FM + CCU; CS: FM + CCU + SR; LM: large macroaggregate; SM: small macroaggregate; MI: microaggregate; SC: silt and clay; BS: bulk soil.

## Data Availability

The original contributions presented in the study are included in the article and the Supplementary Materials; further inquiries can be directed to the corresponding authors.

## Supplementary Materials

The following supporting information can be downloaded at https://www.mdpi.com/article/10.3390/plants15193044/s1, Figure S1: Soil water content under different treatments at different growth stages in 2019 and 2020. Vertical bars indicate standard errors (n = 3). Different lowercase letters denote significant differences among treatments at *p* < 0.05. CK: FM + no fertilization; CU: FM + CCU; CS: FM + CCU + SR; Figure S2: The dynamics of SPAD values at different growth stages under different treatments in 2019 and 2020, with the results of the mixed-effects model embedded. The linear mixed-effects model was analysed with treatment, year, growth stage, and their interactions as fixed effects, and block as a random effect. Growth stage was treated as a repeated-measures factor within each plot. The covariance structure was AR(1), selected by AIC. Denominator degrees of freedom were estimated using the Satterthwaite approximation. Post hoc comparisons were made with the LSD test at *p* < 0.05. Vertical bars indicate standard errors (n = 3). CK: FM + no fertilization; CU: FM + CCU; CS: FM + CCU + SR; Figure S3: Exploratory relationship between grain yield and small macroaggregate N stock (SM N stock) across CU and CS treatments in 2019 and 2020 (n = 12). Partial correlation analysis indicated that the association remained significant after controlling for treatment (r = 0.801, *p* = 0.003) but was attenuated after controlling for year (r = 0.596, *p* = 0.053). CU: FM + CCU; CS: FM + CCU + SR; Table S1: Soil bulk density in various soil layers (0–200 cm) of spring maize in each treatment; Table S2: Nitrogen concentrations in different plant organs at various growth stages of spring maize in each treatment in 2019 and 2020; Table S3: Tests of fixed effects for SWS in 0–40 cm soil layer, SWS in 0–200 cm soil layer, dry matter accumulation, and plant N accumulation under different treatments, years, and growth stages; Table S4: The fixed-depth N stocks for soil aggregate fractions and bulk soil under CU and CS treatments; Table S5: Partial correlation coefficients between crop N parameters and soil aggregate N parameters under CU and CS treatments.
