# Supplementary material for: Film Mulching with Straw Return and Controlled-Release and Conventional Urea Blend Alters Aggregate-Associated Nitrogen Distribution While Sustaining Grain Yield in Dryland Maize
Source: Plants (Basel). 2026 Oct 5;15(19):3044. doi: 10.3390/plants15193044 (PMC13666776; doi:10.3390/plants15193044)
Supplement: Supplementary file 1 [file plants-15-03044-s001.zip › plants-4569000-supplementary.pdf]

Supplementary Material

Film Mulching with Straw Return and Controlled-Release and Conventional Urea Blend Alters Aggregate-Associated Nitrogen Distribution While Sustaining Grain Yield in Dryland Maize

Fangfang Zhang [1,2], Yixuan Lv [1], Yanting Lu [1], Ziyi Song [1], Xuedong Chen [1], Kongyang Wu [1], Qilong Song [2,3,*] and Renshan Li [1,*]

[1] College of Life Science, Luoyang Normal University, Luoyang 471934, China; setzhang@126.com (F.Z.); 19930766043@163.com (Y.L.); 18638360792@163.com (Y.L.); 13782479599@163.com (Z.S.); chenxuedong1224@lynu.edu.cn (X.C.); kywu2007@lynu.edu.cn (K.W.)

[2] State Key Laboratory of Soil and Water Conservation and Desertification Control, Northwest A&F University, Xianyang 712100, China

[3] College of Resources and Environmental Sciences, Gansu Agricultural University, Lanzhou 730070, China

* Correspondence: songql@nwafu.edu.cn (Q.S.); rsli@lynu.edu.cn (R.L.)

Table S1. Soil bulk density in various soil layers (0–200 cm) of spring maize in each treatment.

Soil Layer	CK	CU	CS
cm		g cm⁻³	
0-20	1.37±0.06a	1.40±0.02a	1.32±0.08a
20-40	1.41±0.05b	1.44±0.04ab	1.50±0.03a
40-60	1.49±0.08a	1.43±0.03a	1.43±0.01a
60-80		1.31	
80-100		1.30	
100-120		1.27	
120-140		1.26	
140-160		1.25	
160-180		1.28	
180-200		1.29	

Values are means ± standard error (n = 3). Different lowercase letters denote significant differences among treatments at $P < 0.05$. Values below 60 cm depth are means across treatments, because bulk density at these depths did not differ among treatments.

Table S2. Nitrogen concentrations in different plant organs at various growth stages of spring maize in each treatment in 2019 and 2020.

Stages	Treatments	Nitrogen Concentration (g kg^{-1})					
		2019			2020		
		CK	CU	CS	CK	CU	CS
V6	Leaf	28.8±2.6	35.3±1.9	36.8±1.1	23.8±0.8	38.5±0.4	37.3±1.2
V10	Stem	11.3±0.4	14.7±3.2	16.5±3.3	6.7±0.3	15.4±0.5	19.3±1.4
	Leaf	17.6±0.9	24±2.3	22.2±1.4	18.5±1.1	32.7±1	30.3±0.2
R1	Stem	4.3±0.1	8.5±0.1	7.1±0.8	4.1±0.5	11.2±0.7	9.3±0.4
	Leaf	8.5±0.4	24.2±0.8	25.5±0.6	10.9±0.9	25.6±2.1	31±2.6
	Bract	15.9±0.8	18.1±0.6	17.4±0.8	14.3±1.1	14.3±0.9	17±0.3
R3	Stem	1.1±0.2	3.6±0.3	2.3±1	2.0±0.2	5.7±0.3	5.7±0.2
	Leaf	10±0.2	22.4±1.9	24.5±0.5	12.0±0.4	25.2±1.1	24.1±0.5
	Bract	7.8±0.9	5.7±0.3	3.9±1.7	5.7±0.5	7.1±0.8	6.5±0.1
	Cob	6.8±0.3	5.5±1.1	4.8±1.1	11.5±0.3	16±3.1	5.8±2.2
	Kernel	10.4±0.4	14±0.4	16±0.8	5.3±0.5	18.2±0.9	16.4±0.4
R6	Stem	2.4±0.2	3.2±0.1	3.5±0.2	3.4±0.2	4.0±0.3	4.1±0.1
	Leaf	8.3±0.1	11.2±0.6	12.4±0.1	10.2±0.4	16.8±1.1	19.6±0.7
	Bract	2.2±0.2	2.4±0.1	2.4±0.1	5.0±0.4	4.3±0.7	5.3±0.2
	Cob	4.9±0.1	4.5±0.2	4.4±0.2	5.6±0.1	6.3±0.8	5.6±3.1
	Kernel	9.1±0.2	12.3±0.2	13.2±0.1	9.4±0.6	14.2±0.5	17.2±0.2

Table S3. Tests of fixed effects for SWS in 0–40 cm soil layer, SWS in 0–200 cm soil layer, dry matter accumulation, and plant N accumulation under different treatments, years, and growth stages.

**Source of Variation**	**SWS (0–40 cm)**			**SWS (0–200 cm)**			**Dry matter Accumulation**			**Plant N Accumulation**		
**-**	**df**	**F**	**P**	**df**	**F**	**P**	**df**	**F**	**P**	**df**	**F**	**P**
Treatment (T)	2	18.314	<0.0001	2	33.364	0.001	2	313.025	<0.0001	2	756.442	<0.0001
Year (Y)	1	9.655	0.003	1	2.232	0.146	1	42.710	<0.0001	1	0.72	0.399
Stage (S)	5	231.028	<0.0001	5	324.969	<0.0001	4	1352.226	<0.0001	4	392.391	<0.0001
T × Y	2	3.53	0.034	2	2.549	0.096	2	0.053	0.949	2	4.622	0.014
T × S	10	6.335	<0.0001	10	7.307	<0.0001	8	89.181	<0.0001	8	56.05	<0.0001
T × Y × S	15	31.435	<0.0001	15	59.43	<0.0001	12	4.267	<0.0001	12	1.707	0.088

Table S4. The fixed-depth N stocks for soil aggregate fractions and bulk soil under CU and CS treatments.

Treatments	Aggregate N Stock (Mg ha^{-1})				
	**LM**	**SM**	**MI**	**SC**	**BS**
2019					
CU	0.55b	1.5a	0.53a	0.24a	2.67a
CS	0.88a	1.55a	0.34b	0.19a	2.96a
2020					
CU	0.49a	1.37a	0.63a	0.33a	2.65a
CS	0.86a	1.48a	0.30b	0.19a	2.75a
**Source of variation**					
Year (Y)	ns	ns	ns	ns	ns
Treatment (T)	**	ns	**	ns	ns
Y×T	ns	ns	ns	ns	ns

Different lowercase letters denote significant differences among treatments in the same year at $P < 0.05$. *, **, ***, ns indicate significant differences at $P < 0.05$, $P < 0.01$, $P < 0.001$, and no significance, respectively. LM: Large macroaggregate; SM: Small macroaggregate; MI: Microaggregate; SC: Silt and clay; BS: Bulk soil.

Table S5. Partial correlation coefficients between crop N parameters and soil aggregate N parameters under CU and CS treatments.

		LM	SM	MI	SC	LM Stock	SM Stock	MI Stock	SC Stock	TN Stock	Straw N_{conc}	Straw N_{acc}	Total N_{up}	Yi-eld
**Control**	LM	1												
**Variable:**	SM	0.045	1											
**Year**	MI	-0.778**	-0.503	1										
	SC	-0.529	-0.433	0.297	1									
	LM stock	0.971***	0.106	-0.811**	-0.475	1								
	SM stock	0.176	0.701*	-0.557	-0.146	0.362	1							
	MI stock	-0.748**	-0.49	0.985***	0.268	-0.76**	-0.457	1						
	SC stock	-0.501	-0.341	0.229	0.973***	-0.405	0.028	0.223	1					
	TN stock	0.003	0.855**	-0.399	-0.334	0.023	0.553	-0.377	-0.239	1				
	straw N_{conc}	0.309	0.146	-0.069	-0.567	0.356	0.284	0.026	-0.408	0.252	1			
	straw N_{acc}	0.485	0.28	-0.478	-0.336	0.485	0.393	-0.424	-0.28	0.436	0.392	1		
	Total N_{up}	0.79**	0.09	-0.561	-0.552	0.753**	0.135	-0.522	-0.483	0.151	0.638*	0.633*	1	
	Yield	-0.533	0.547	0.061	0.236	-0.372	0.596	0.076	0.362	0.396	0.092	-0.04	-0.308	1
**Control**	LM	1												
**Variable:**	SM	-0.822**	1											
**Treatment**	MI	-0.083	-0.143	1										
	SC	-0.099	-0.187	-0.618*	1									
	LM stock	0.812**	-0.692*	-0.169	0.082	1								
	SM stock	-0.611*	0.604*	-0.224	0.242	-0.125	1							
	MI stock	-0.048	-0.171	0.931***	-0.534	-0.138	-0.1	1						
	SC stock	-0.092	-0.167	-0.513	0.88***	-0.025	0.226	-0.314	1					
	TN stock	-0.891***	0.8**	0.127	-0.059	-0.912 ***	0.378	0.1	0.004	1				
	straw N_{conc}	-0.288	0.103	0.258	-0.009	0.13	0.325	0.037	-0.378	0.008	1			
	straw N_{acc}	-0.237	0.078	0.155	0.093	0.056	0.313	0.002	-0.233	0.129	0.748**	1		
	Total N_{up}	-0.027	-0.172	0.3	-0.013	0.261	0.025	0.06	-0.377	-0.232	0.893***	0.747**	1	
	Yield	-0.772**	0.735*	-0.172	0.192	-0.433	0.801**	-0.137	0.254	0.548	0.205	0.168	-0.015	1

LM: > 2 mm; SM: 0.25–2 mm; MI: 0.053–0.25 mm; SC: < 0.053 mm; LM N stock: the N stock in LM fraction; SM N stock: the N stock in SM fraction; MI N stock: the N stock in MI fraction; SC N stock: the N stock in SC fraction; BS N stock: the N stock in bulk soil; total N_{up}: the total N uptake; straw N_{conc}: the N concentration of the returned straw; straw N_{acc}: the N accumulation of the returned straw. *, **, *** indicate significant correlation at $P < 0.05$, $P < 0.01$, $P < 0.001$, respectively.

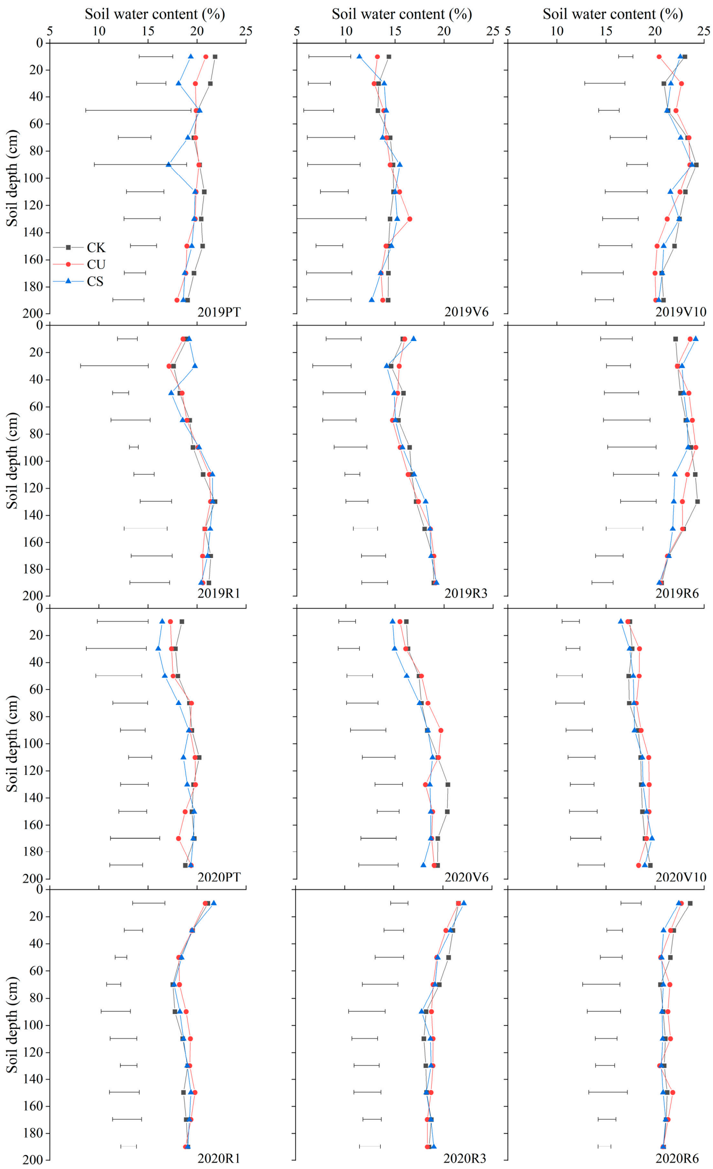

Figure S1. Soil water content under different treatments at different growth stages in 2019 and 2020. Vertical bars indicate standard errors (n = 3). Different lowercase letters denote significant differences among treatments at $P < 0.05$. CK: FM + no fertilization; CU: FM + CCU; CS: FM + CCU + SR.

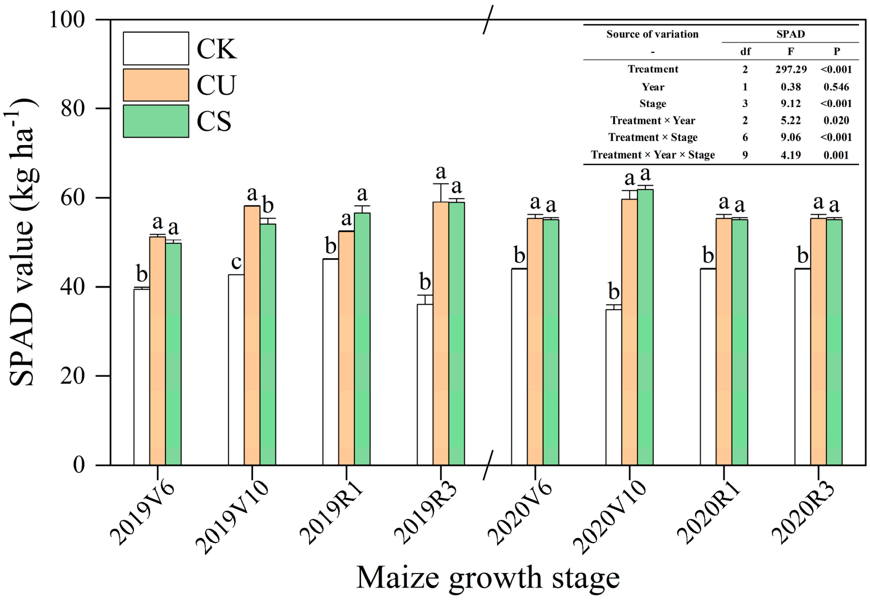

Figure S2. The dynamics of SPAD values at different growth stages under different treatments in 2019 and 2020, with the results of the mixed-effects model embedded. The linear mixed-effects model was analysed with treatment, year, growth stage, and their interactions as fixed effects, and block as a random effect. Growth stage was treated as a repeated-measures factor within each plot. The covariance structure was AR(1), selected by AIC. Denominator degrees of freedom were estimated using the Satterthwaite approximation. Post hoc comparisons were made with the LSD test at $P < 0.05$. Vertical bars indicate standard errors (n = 3). CK: FM + no fertilization; CU: FM + CCU; CS: FM + CCU + SR.

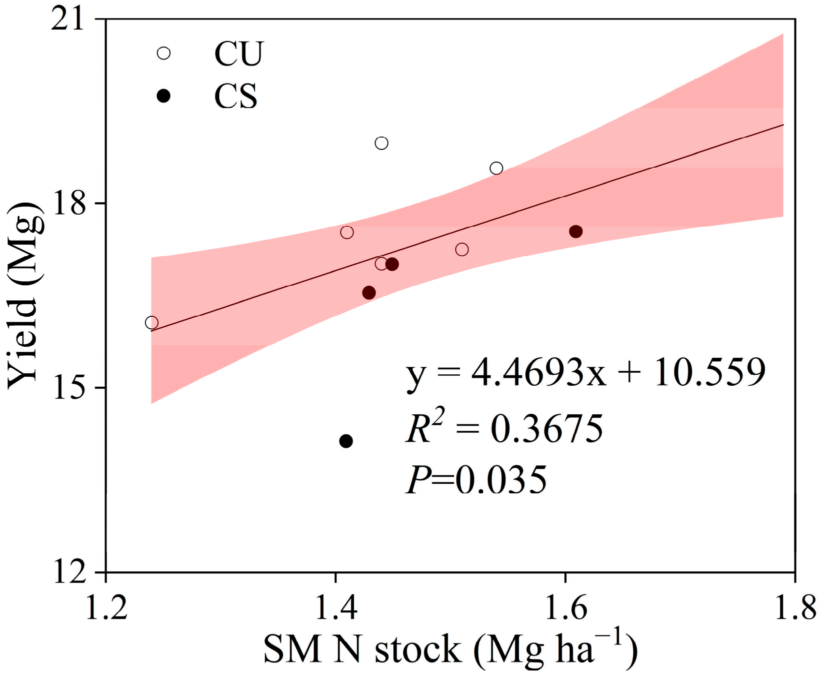

Figure S3. Exploratory relationship between grain yield and small macroaggregate N stock (SM N stock) across CU and CS treatments in 2019 and 2020 (n = 12). Partial correlation analysis indicated that the association remained significant after controlling for treatment (r = 0.801, $P = 0.003$) but was attenuated after controlling for year (r = 0.596, $P = 0.053$). CU: FM + CCU; CS: FM + CCU + SR.
